# Overweight or about right? A norm comparison explanation of perceived weight status

**DOI:** 10.1002/osp4.89

**Published:** 2017-01-25

**Authors:** E. Robinson, I. Kersbergen

**Affiliations:** ^1^ Psychological Sciences University of Liverpool Liverpool UK

**Keywords:** Body image, norms, perceived weight, weight status misperceptions

## Abstract

**Objectives:**

Body‐weight norms may explain why personal evaluations of weight status are often inaccurate. Here, we tested a ‘norm comparison’ explanation of weight status perceptions, whereby personal evaluations of weight status are biased by perceived body‐weight norms.

**Methods:**

Study 1 examined whether perceptions of how one's own body weight compares to an average person predict personal evaluations of weight status. Study 2 examined whether manipulating perceptions of how one's own body weight compares to an average person influences whether or not a person identifies their own weight status as being overweight.

**Results:**

In Study 1, if participants rated their body weight as being similar to the body weight of an average person, they were less likely to identify their weight status as being overweight. In Study 2, participants that were led to believe that their body weight was heavier than the average person were more likely to perceive their own weight status as being overweight.

**Conclusions:**

Personal perceptions of weight status are likely to be shaped by a ‘norm comparison’ process. As overweight becomes more normal, underestimation of weight status amongst individuals with overweight and obesity will be more common.

## Introduction

Weight status misperceptions occur when there is a discrepancy between the weight status a person believes he or she is and their actual weight status. For example, although a substantial proportion of individuals with overweight or obesity believe they are ‘overweight’, a large number underestimate their weight status as being ‘about right’ 1, 2, 3, 4. Likewise, some normal‐weight females incorrectly believe they are ‘overweight’ 4, 5, 6, 7. Thus, personal misperceptions of weight status are common.

The public health relevance of overweight and obesity‐related weight status misperception has received a considerable amount of attention 1, 8, 9, 10. One of the reasons that perceptions of weight status are thought to be of importance is because they are likely determinants of health behaviour. Normal‐weight adolescents who erroneously believe they are ‘overweight’ are more likely to use unhealthy dieting practises and have lower psychological well‐being 11, 12, 13. Amongst individuals with overweight or obesity, although accurate perception of overweight is associated with greater weight loss intentions 2, 14, there are recent studies that suggest that accurate identification of overweight may also be associated with a number of adverse health outcomes, including depressive symptoms and weight gain 11, 15, 16, 17.

Some epidemiological research has suggested that one reason why individuals with overweight or obesity fail to accurately identify their weight status may be because of increases in the prevalence of obesity 18, 19, 20. Increases in obesity may have altered body‐weight norms and resulted in larger sized bodies appearing more normal, which in turn has increased underestimation of weight status. This perspective is in line with the observation that people who are more frequently exposed to heavier body weights are particularly likely to underestimate their weight status 21, 22. Although the proposal that personal perceptions of weight status are influenced by body‐weight norms has been suggested by a number of researchers 4, 18, 20, 22, there has been no direct testing of this hypothesis. In addition, there have been few examinations of the psychological processes that influence personal perceptions of weight status. Research in social psychology suggests that when a person evaluates their own behaviour or appearance, they do so by making social comparisons 23, 24. Thus, we propose that when a person evaluates their own weight status, they are influenced by how ‘normal’ they believe their body weight is compared with others. Because the ‘average’ body size of an adult is now classed as being overweight in many countries, this ‘norm comparison’ explanation of perceived weight status could be responsible for why a large number of individuals with overweight and obesity underestimate their weight status.

The aim of the present research was to test a ‘norm comparison’ explanation of perceived weight status. More specifically, we tested the notion that when a person decides whether they are overweight or not, they are strongly influenced by how they perceive their body weight compares to that of an ‘average’ man or woman. In Study 1, we tested this by examining whether participants' perceptions of how their own weight corresponds to an average person's body weight are predictive of self‐perceived weight status in a cross‐sectional design. In Study 2, we experimentally manipulated whether participants believed their body weight was heavier or slimmer than average and examined if this influenced their personal evaluations of weight status.

## Study 1

In Study 1, we tested whether men and women's perceptions of how their own body weight corresponds to others may be an important determinant of self‐perceived weight status. As different forms of social comparison can influence judgements 28, 29, we measured participants' perceptions of how their own body weight corresponded to a number of social reference points; comparison to an average person, body weight compared with all others, comparison to slim others and comparison to heavy others.

### Methods

#### Data collection

Both studies 1 and 2 were conducted using Amazon Mechanical Turk (MTURK). MTURK is an online participant recruitment resource that has been shown to be a valid data collection source 25 and has previously been used in psychological and obesity‐related research 26, 27. In both studies, participation was in exchange for a small monetary reward and the authors' institutional ethics review board approved the study procedures.

#### Participants

One hundred and twenty one (60 women, 61 men) US participants (M age = 31.1 years old, SD = 10.4) were recruited into an online survey study about ‘personal characteristics’ using MTURK. Mean body mass index (BMI) (self‐reported weight/height^2^) = 27.0, SD = 7.3.

#### Procedure

On three consecutive pages of the online survey (counter‐balanced), participants self‐reported their current weight and height, their beliefs about how their body weight corresponded to other men/women and their self‐perceived weight status. Based on 1, 2, 3, 4, 5, 6, 7, self‐perceived weight status was measured by asking participants ‘what would you describe your weight as?’, and participants provided their response on a 100‐point visual analogue scale with anchors: very underweight to very obese. Participants answered four questions in a counterbalanced order about how their body weight compared with others of the same sex. We self‐devised the social comparison measures as these constructs have not been studied previously. To measure comparison to an average‐sized person, participants rated ‘compared to an average male/female, I am’ (1–9 response format, 1 = a lot slimmer, 9 = a lot heavier). To measure beliefs about how body weight ranked relative to all other men/women, participants rated ‘in relation to all males/females of my age, I am’ (1 = heavier than all other men/women, 9 = slimmer than all other males/females). To measure beliefs about how body weight related to the slimmest men/women, participants were asked ‘compared to the very slimmest males/females of my age, I am’ (1 = the same weight, 9 = a lot heavier). To measure beliefs about how body weight related to the heaviest men/women, participants were asked ‘compared to the very heaviest males/females of my age, I am’ (1 = a lot slimmer, 9 = the same weight). Participants completed demographic information and were then debriefed. We used different response formats for the self‐perceived weight status rating versus social comparison measures to reduce shared measurement variance.

### Results

See Table 1 for participant characteristics. We used forced entry linear regression analysis to examine whether the four measures of social comparison predicted self‐perceived weight status rating whilst controlling for demographic variables (e.g. BMI, gender, age, ethnicity, annual income and highest level of education). We controlled for demographic variables in our main analysis because previous research has reliably shown that demographic factors are associated with personal perceptions of weight status 2, 19, and therefore, they could act as potential confounders if not controlled for. The overall model was significant (Adjusted *R* ^2^ = 0.81, *p* < .001). Being of higher BMI and female were predictive of a heavier self‐perceived weight status rating, whereas ethnicity, current income, age and highest level of education were not significant predictors. In relation to our main hypotheses, perceptions of how one's body weight compared with an average man/woman was a significant predictor of self‐perceived weight status rating, whereby believing that one's body weight was larger than average was associated with a heavier self‐perceived weight status rating. Perceptions of how one's body weight ranked compared with all others and perceptions of how one's body weight compared with heavy others were not significant predictors (Table 2). Comparison to slim others was a significant predictor of self‐perceived weight status ratings, whereby believing that one's body weight was heavier than the slimmest males/females was predictive of a heavier perceived weight status rating. We also examined whether the same results were observed when using stepwise (as opposed to forced entry regression models), and this was the case. In addition, we also examined whether any of the social comparison variables interacted with participant BMI to predict self‐perceived weight status ratings in a further set of regression models. There were no significant interactions, indicating that the association observed between perceptions of how one's body weight compares to others and self‐perceived weight status is similar in participants of higher and lower BMI.

**Table 1:** Participant characteristics in Study 1

Number of participants	121
Age (years, SD)	31.1 (10.4)
BMI (mean self‐reported weight/height^2^, SD)	27.0 (7.3)
Gender	61 M, 60 F
Ethnicity (% white)	72.7%
Highest level of education (mean, SD)	3.8 (1.3)
Current annual income (mean, SD)	2.6 (1.5)

Highest level of education: 1 = did not complete high school; 2 = high school; 3 = some college; 4 = bachelor's degree; 5 = master's degree; 6 = doctoral or professional degree.

Current annual income: 1 = less than $25,000 (student); 2 = less than $25,000 (non‐student); 3 = $25–39,999; 4 = $40–49,999; 5 = $50–74,999; 6 = $75–99,999; 7 = $100,000 or higher.

BMI, body mass index; M, male; F, female.

**Table 2:** Predictors of self‐perceived weight status in Study 1

	Standardized *β*	*p* value
Model including all participants		
Comparison to average person	.30	.004*
Rank comparison	−.05	.35
Comparison to heavy others	.08	.24
Comparison to slim others	.21	.001*
Model including only male participants		
Comparison to average person	.76	<.001*
Comparison to slim others	.15	.10
Model including only female participants		
Comparison to average person	.51	<.001*
Comparison to slim others	.42	<.001*

See text for rating scales.

* Statistically significant predictor at *p* < .05.

Given that female body image is thought to be particularly influenced by portrayals of the thin ideal 30, 31, we reasoned that the comparison to slim others effect we observed may have been driven by female participants. To test this, we conducted a further forced entry regression model and computed interaction terms between gender and comparison to slim others and between gender and comparison to an average person, which were entered alongside gender, comparison to slim others and comparison to an average person. In this model, the main effect of comparison to an average person remained significant (standardized *β* = .71, *p* < .001), comparison to slim others was no longer significant (standardized *β* = .14, *p* = .10), the interaction between gender and comparison to an average person was not significant (standardized *β* = −.13, *p* = .19), but the gender interaction with comparison to slim others was significant (standardized *β* = .20, *p* = .048). To follow up this interaction, we used separate regression models to examine whether comparison to an average person and comparison to slim others predicted self‐perceived weight status ratings in male and female participants separately. Amongst men, comparison to an average male was a significant predictor and comparison to slim men was not. Amongst women, both comparison to an average woman and comparison to slim women were significant predictors (Table 2).

### Conclusions

Study 1 indicated that personal evaluations of weight status may be influenced by perceptions of how one's body weight compares to an ‘average’ person. Although these data were in fitting with our hypotheses, Study 1 used a cross‐sectional design, and this does not allow for causal inference. We addressed this limitation in Study 2.

## Study 2

The aim of Study 2 was to experimentally manipulate participants' beliefs about whether they are heavier or slimmer than average and examine the effect this had on self‐perceived weight status. We did this by providing participants with bogus feedback about how their body weight compared with others of the same gender, ethnicity and age range. To be able to do so convincingly, we aimed to recruit participants we presumed would be most likely to believe bogus information, which suggested that they were either heavier or slimmer than average (i.e. those with a BMI close to the cut off for the overweight range, BMI = 25).

### Methods

#### Participants

After visiting the online study site, potential participants were asked to report their weight and height, gender, age range and ethnicity. If a participant's weight and height responses indicated a BMI outside of the range 23–27.9, on the next page they were instructed that they were ineligible for the study and thanked for their time. One hundred and fifty‐two (50 women, 72 men) US participants (median age range = 18–29 years old) were recruited into a survey study about ‘effective public health communication’ via MTURK. Mean BMI (self‐reported weight/height^2^) = 25.2, SD = 1.4.

#### Procedure

To disguise the study aims, participants were informed that the study concerned developing effective ways of communicating public health information about body weight and that they would be asked to provide feedback on graphical presentations of how their body size compares to other members of the population. Participants were randomized to receive four different graphics that either indicated that they were heavier (heavier than average condition) or slimmer (slimmer than average condition) than other people from the same age range, gender and ethnicity. The first graphic was of a range of nine body‐size silhouettes in ascending order with an arrow indicating an ‘average person's’ weight of the same age range and gender (between the fourth and fifth silhouette) and a second arrow indicating that the participant's weight placed them one silhouette above or below that average (dependent on condition). The remaining graphics were of a pie chart, a population distribution graph and written text indicating that the participant's body weight was heavier or slimmer than average. To corroborate the cover story, underneath each of the graphics, participants were asked to rate the clarity, appearance and their comprehension of the images and information, as well as being provided with a free‐text box for further comments.

To corroborate the survey study cover story, participants were asked to provide personal information such as how often they smoked and drank alcohol, as well as the self‐perceived weight status rating; what would you describe your weight as? (100‐point visual analogue scale, anchors: very underweight to very obese, as used in Study 1) and further demographics. On the next page, embedded within a series of questions about how aspects of their behaviour compared with their peers, we measured self‐perceived weight normality. We asked participants ‘compared to the average male/female of my age, I am’ (1–9 scale, anchors: a lot slimmer, a lot heavier). On the next page, participants were asked to provide their overall opinions about the graphics and information presented (e.g. do you think others would find this information useful?). Within this section, we included two items that acted as a manipulation credibility check and measured how believable participants thought the bogus feedback was ‘Overall, do you think the text and images about your weight were believable/accurate for you?’, 5‐point scale, not at all to extremely. Participants were asked to guess the aims of the study and then debriefed.

#### Manipulation check

Five participants came close to guessing the aims of the study, although their exclusion from analyses has no effect on any of the results reported. Because we predicted that whether participants believed the bogus feedback was credible or not would affect the success of our manipulation, we formally assessed credibility of the bogus feedback. We summed the two items measuring believability and accuracy as they were highly correlated (*r* = .81, *p* < .001). Participants who scored below the midpoint of this scale (<3 on a 1–5 scale) were classed as having rejected the bogus feedback (*n* = 51), whilst participants scoring at the midpoint or above were classed as having believed the bogus feedback was credible (*n* = 101), resulting in two distinct sub‐groups of participants. A 2 × 2 ANOVA (factors: condition and credibility) on the weight normality measure indicated a significant interaction between condition and credibility (*F*(1, 148) = 17.5, *p* < .001, *np* ^*2*^ = .11). Independent samples *t*‐tests showed that the manipulation was successful amongst participants who believed the feedback was credible; participants in the heavier than average condition were more likely to believe they were heavier than other people, in comparison to participants in the slimmer than average condition (*t* (99) = 8.2, *p* < .001, *d* = 1.6) (Table 3). As expected, the manipulation was unsuccessful amongst those who rejected the bogus feedback; there was no difference (*t* (49) = .10, *p* = .93, *d* = 0.03) between participants in the heavier than average (M = 5.3, SD = 1.4) and the slimmer than average (M = 5.3, SD = 1.6) conditions.

**Table 3:** Perceived normality and weight status perception by condition for participants believing the bogus feedback was credible in Study 2

	Perceived normality^a^	Weight status perception^b^
Heavier than average feedback condition (*n* = 44)	6.3.(0.8)*	52.4 (14.6)*
Slimmer than average feedback condition (*n* = 57)	4.4 (1.4)*	44.6 (13.4)*

a ‘Compared to the average male/female of my age, I am’ (1–9 scale, anchors: a lot slimmer, a lot heavier).

b ‘How would you describe your weight?’ (100‐point visual analogue scale, anchors: very underweight to very obese).

* Significant difference at *p* < .05 between conditions. Values are means (SDs).

### Results

See Table 4 for participant characteristics. To examine the effect of manipulating perceived averageness of body weight, we compared the effect of experimental condition on self‐perceived weight status ratings amongst participants who believed the bogus feedback was credible (*n* = 101). Participants who had been led to believe that their body weight was above average had a significantly heavier self‐perceived weight status rating (*t* (99) = 2.8, *p* < .001, *d* = 0.56) than participants led to believe they were slimmer than an average person (Table 3). As expected, perceived normality of weight and self‐perceived weight status ratings were significantly correlated (*r* = .51, *p* < .001), so we next tested mediation. In line with our hypotheses, mediation analysis confirmed that the effect of experimental condition on self‐perceived weight status rating was mediated by changes to perceived normality of body weight; using a bootstrapping procedure 32, the indirect effect was statistically significant (indirect effect = 10.4, 95% CIs: 15.93, 5.86).

**Table 4:** Characteristics of participants used in analyses for Study 2

Number of participants	101
Age (% aged 18–29)	51.5%
BMI (mean self‐reported weight/height^2^, SD)	25.3 (1.4)
Gender	73 M, 28 F
Ethnicity (% white)	71.3%
Highest level of education (mean, SD)	4.2 (1.4)
Current annual income (mean, SD)	3.1 (1.7)

We examined whether the two experimental conditions in Study 2 differed for any of the above demographic variables and they did not (*p* > .05)

Highest level of education: 1 = did not complete high school; 2 = high school; 3 = some college; 4 = bachelor's degree; 5 = master's degree; 6 = doctoral or professional degree.

Current annual income: 1 = less than $25,000 (student); 2 = less than $25,000 (non‐student); 3 = $25–39,999; 4 = $40–49,999; 5 = $50–74,999; 6 = $75–99,999; 7 = $100,000 or higher.

BMI, body mass index; M, male; F, female.

Given the results of Study 1, we also tested whether gender moderated the effect of feedback condition on self‐perceived weight status ratings (2 × 2 ANOVA); the interaction between gender and condition was not significant (*F* (1, 97) = 0.14, *p* = .71, *np* ^*2*^ *= .001*), suggesting that our results were consistent across men and women. Although we used an experimental design with random assignment, to further test robustness, we also examined whether our main results remained the same when controlling for demographic variables (e.g. participant BMI, gender, age, income and education level) and this was the case.

## General discussion

In Study 1, we examined whether perceptions of how one's own body weight compares to an ‘average’ person predicts personal evaluations of weight status. If participants believed that their body weight was relatively ‘normal’ or slimmer than average, they were less likely to identify their weight status as being overweight. In Study 2, we examined whether experimentally manipulating perceptions of how one's own body weight compares to an ‘average’ person influences personal evaluations of weight status. Results indicated that participants led to believe their body weight was slimmer, as opposed to larger than an average person, were less likely to identify their weight status as being ‘overweight’.

The proposal that weight status evaluations may be skewed or biased by body weight norms has been suggested by a number of researchers 18, 20, 33, 34, but not directly examined. The present studies provide direct experimental evidence in support of this hypothesis. The results of the present studies are also in fitting with social comparison theories of judgement 23, 24. We propose that when individuals evaluate their own weight status, they do so by comparing their body size to their internal representation of what an ‘average’ sized body is.

This ‘norm comparison’ account may also explain why increases in obesity have been associated with fewer individuals with overweight or obesity identifying their weight status as being ‘overweight’ or ‘too heavy’. A person's internal representation of what constitutes a ‘normal’‐sized body is likely to be shaped by the types of bodies they frequently encounter 35, 36, 37, so as obesity becomes more prevalent, perceptions of a ‘normal’ sized body will become larger. The proposed norm comparison explanation of perceived weight status may also be useful in explaining why there are socioeconomic and ethnic differences in weight perception. Individuals from poorer areas and of Black or Hispanic background are more likely to live in areas of high obesity prevalence and are less likely than their richer white counterparts to identify that they are overweight or overestimate their weight status 38, 39, 40. This may be because their internal representation of what constitutes a normal‐sized body is larger due to encountering heavier body weights more frequently.

Further understanding of the types of comparisons individuals use to make weight status judgements would now be valuable. Our findings suggest that perceptions of what constitutes an ‘average’ body weight may be of particular importance. However, research in other domains suggests that individuals will sometimes base self‐evaluations on other types of social comparison rather than simple ‘averageness’, such as upwards and downwards social comparisons 28, or relative rank position in a population 29. In Study 1, we found some evidence that how women (but not men) felt their body compared to very thin others predicted their self‐perceived weight status. One interpretation of this finding is the internalization of the ‘thin ideal’, whereby females may be more likely than males to assess the appropriateness of their own body weight relative to slender women because of the value and importance attached to being feminine and thin. This may also in part explain why overestimation of weight status tends to be more common amongst women than men 4, 7.

Although our focus here was on perceived normality of body weight and weight status judgements, future work would also benefit from examining the importance of other processes shaping weight status evaluations made about the self and others. Labels such as ‘overweight’ and ‘obese’ are likely to be viewed negatively by some, so it is conceivable that this could promote underestimation of weight status amongst individuals with overweight. In addition, both how ‘normal’ one's own body size is perceived to be and self‐perceived weight status may be in part determined by physical characteristics like body fat distribution 41, so further work examining this would now be valuable. The relevance that the present findings have for other research on body image in obesity may now be interesting to examine. Although we know that obesity tends to be linked with poor body image 42, 43, 44, not all individuals with overweight or obesity will have low body dissatisfaction or negative body image 42. One factor that may be important in explaining this is the extent to which an individual believes their body size deviates from normality; until a person believes their body size deviates from normality and is ‘overweight’, negative body image and body dissatisfaction would presumably be rare. In addition, given that weight status is often misperceived and skewed by body‐weight norms, intervention work to correct perceptions of weight status may be beneficial. Addressing unrealistic body weight norms and reducing overestimation of weight status amongst young women may be one such application. In a similar vein, it could be argued that ensuring overweight individuals are aware of their weight status will encourage greater weight management efforts, but whether this would produce positive outcomes is less clear. There is stigma attached to being ‘overweight’, and recent findings suggest that individuals who perceive their weight status as being ‘overweight’ are counter‐intuitively more likely to gain further weight than those who do not identify as being overweight 6, 15, 16. Thus, intervention work to address perceptions of weight status will need to consider these findings carefully.

There were limitations to our studies. We sampled White US participants predominantly. Studies designed to examine whether other demographic groups show a similar pattern of results would now be valuable. To examine how participants believed their body size related to other people, we had to make use of self‐devised measures and it will be important in future work to assess their validity and reliability. In both studies, we measured self‐reported BMI as a proxy measure of body weight, as opposed to objectively measured BMI. Self‐reported BMI is prone to error. However, given that our main hypotheses examined the relationship between perceived normality of weight and weight status evaluations, and Study 2 used an experimental manipulation, it is unlikely that the error associated with self‐reported BMI will have affected any of the main conclusions of the present work.

## Conclusions

Personal perceptions of weight status are likely to be shaped by a ‘norm comparison’ process. As overweight becomes more ‘normal’, underestimation of weight status amongst individuals with overweight and obesity will be more common.

## Funding

This research received no external funding. ER was supported by the Medical Research Council.

## Conflict of Interest Statement

The authors declared that they had no conflicts of interest with respect to their authorship or the publication of this article.
